# Leadership in healthcare education

**DOI:** 10.1186/s12909-020-02288-x

**Published:** 2020-12-03

**Authors:** Christie van Diggele, Annette Burgess, Chris Roberts, Craig Mellis

**Affiliations:** 1 The University of Sydney, Faculty of Medicine and Health, The University of Sydney, Edward Ford Building A27, Sydney, NSW 2006 Australia; 2 The University of Sydney, Faculty of Medicine and Health, Sydney Health Professional Education Research Network, The University of Sydney, Sydney, Australia; 3 The University of Sydney, Faculty of Medicine and Health, Sydney Medical School – Education Office, The University of Sydney, Sydney, Australia; 4 The University of Sydney, Faculty of Medicine and Health, Sydney Medical School – Central Clinical School, The University of Sydney, Sydney, Australia

**Keywords:** Leadership, Leadership theory, Teamwork, Role models, Management, Organisational goals

## Abstract

Effective leadership is a complex and highly valued component of healthcare education, increasingly recognised as essential to the delivery of high standards of education, research and clinical practice. To meet the needs of healthcare in the twenty-first century, competent leaders will be increasingly important across all health professions, including allied health, nursing, pharmacy, dentistry, and medicine. Consequently, incorporation of leadership training and development should be part of all health professional curricula. A new type of leader is emerging: one who role models the balance between autonomy and accountability, emphasises teamwork, and focuses on improving patient outcomes. Healthcare education leaders are required to work effectively and collaboratively across discipline and organisational boundaries, where titles are not always linked to leadership roles. This paper briefly considers the current theories of leadership, and explores leadership skills and roles within the context of healthcare education.

## Background

Leadership has many interpretations, and has been likened to “*the abominable snowman whose footprints are everywhere but who is nowhere to be seen”* [1]. It is an influential process, through which groups of people work towards the achievement of a common goal [2]. Leaders have the ability to shape and influence their followers’ values, attitudes and behaviours through a dyadic relationship. They are able to gain and enlist the support of others in order to achieve shared goals [3, 4]. Effective leadership is a complex and highly valued component of healthcare education, increasingly recognised as essential to the delivery of high standards of education, research and clinical practice [3]. In order to achieve more effective outcomes, leadership and management skills are now an expectation and requirement in the healthcare education setting [5]. However, leaders within healthcare education should not rely on formal positions of authority, but instead, utilise their own appropriate leadership qualities irrespective of their level within the organisation [3]. A new type of leader is emerging: one who role models the balance between autonomy and accountability, emphasises teamwork, and focuses on improving patient outcomes [3]. This paper briefly considers the theories of leadership, and explores leadership skills and roles within the context of healthcare education.

### Management versus leadership

Management and leadership are considered just as important as each other in accomplishing organisational goals. However, there are differences in the functions of the two roles. Management produces order and consistency, while leadership produces change and movement [2]. Management has the responsibility of organising all elements within the organisation, so that the leader’s vision and goals are successfully achieved. If poor management is in place, then goals cannot be achieved; and if poor leadership is in place, then there is no clear goal or vision to work towards. Leadership is seen as *“setting direction, influencing others and managing change: with management concerned with the marshalling and organisation of resources and maintaining stability”* [6]. These differences are summarised in Table 1 [6, 7].

**Table 1:** Leadership versus Management (adapted from Swanwick & McKimm, 2011) [6]

Leadership	Management
**● Establishes direction**	**● Plan and Budget**
**-** Creates a shared vision	**-** Resource allocation
**-** Identifies the bigger picture	**-** Time management and process steps
**-** Sets goals and strategies in place	**-** Establish agendas
**● Connecting people**	**● Employment and organising**
**-** Communication of goals	**-** Maintain structure
**-** Team building and networking	**-** Staffing placements
**-** Aims for commitment	**-** Enforce rules and procedures
**● Motivate and Drive**	**● Control and Problem solve**
**-** Inspire and motivate	**-** Reward systems
**-** Empower followers	**-** Identifies problems
**-** Identify and work towards needs	**-** Solves problems/takes corrective actions

#### Transactional and transformational leadership

Leadership is a social construct, and there are many different leadership models [6]. Two broad types of leadership are identifiable: *“transactional”* and *“transformational”.* And their respective features are a useful way to think about the many types of leadership. Transactional and transformational leadership models are normally amalgamated within organisations to “empower others” (transformational) while holding individuals “accountable” (transactional) for their actions [7–9]. While it is clear that both transformational and transactional leadership paradigms are needed for an organisation to be effective, the optimal leader predominantly practices the transformational aspects of leadership, rather than transactional [10].

### Transactional leadership

The transactional model is seen as an authoritative relationship that is transaction based, where exchanges occur between a leader and follower, once specific goals are identified or decided upon. Transactional leaders value order and structure, and have formal authority, with positions of responsibility within organisations. They achieve organisational goals through a rewards system and through positive reinforcement. A weakness of this model is the lack of innovation, as individuals are driven by predetermined outcomes, and there is lack of incentive and motivation to perform beyond what is expected [6].

### Transformational leadership

Since the introduction of transformational leadership, the concept of leadership has undergone a major shift from representing an authoritative relationship (transactional), to a process of influencing individuals (transformational). Transformational leadership involves leadership through the transformation of individuals or ‘followers’, to work towards a common organisational goal [9–11]. This contemporary form of leadership is based on inspiring individuals, and forming teams to achieve goals. *Transformational* leaders define organisations through the articulation of a clear vision and clear values. The four “I”s of transformational leadership are outlined in Table 2 [9].

**Table 2:** The four “I”s of transformational leadership (adapted from Bass & Aviolo, 1994) [9]

**Idealised influence**	
Pride, respect and trust is stimulated through the development of a vision	
**Inspirational motivation**	
High expectations are created through role modelling	
**Individualised consideration**	
Respect and responsibility is fostered through personal attention to followers	
**Intellectual stimulation**	
New ideas and approaches are used to challenge followers	

#### Team leadership

More recently, the focus has shifted towards *“team leadership”*, with distributed leadership becoming more prevalent within healthcare education, where different professions share influence [12, 13]. Increasingly, leadership involves a collaborative role, with an emphasis on shared leadership and thoughtful allocation of responsibilities. Team-based organisations shift central control from the one leader, to the team. Teams are comprised of members who are interdependent, needing to coordinate their activities in order to accomplish their shared goals [14, 15]. Personal autonomy, accountability, appropriate recognition, and clarity of roles, are all elements that contribute to optimal team performance. However, to ensure success, the organisational culture needs to support the involvement of individuals in these teams, and encourage leadership qualities [15]. Teams often fail when they exist in a traditional authority structure, where organisational culture is not supportive of collaborative work, and lower level decision making. Distributed leadership entails sharing of influence by team members, who step forward, or take a step back as needed. Leadership is provided by the person who meets the specific needs of the team at the time, hence providing faster responses to more complex issues in today’s organisations [15–17]. Effective leaders have an understanding of the conditions needed for teams to function well. For a team to achieve its potential, the operational roles of its members should be matched to their members’ abilities [18]. Belbin (1991) classified nine roles of team members that contribute to its process and function [19], outlined in Table 3. Importantly, within team leadership, no single team role should be regarded as more important than another. Successful teams thrive on their diversity, drawing from the strengths of each member [13].

**Table 3:** Roles of team members that contribute to its process and function (adapted from Belbin, 1991) [19]

ROLE	DESCRIPTION
**Plant:** the ‘ideas’ person	Thoughtful and creative, but may lack communication skills, and attention to required detail.
**Co-ordinator**: the ‘chairperson’	Co-ordinates the work, rather than undertaking the work. Involves all team-members, and mediates discussion.
**Monitor evaluator**: the ‘critic’	Objectively evaluates everything, and may be perceived as negative.
**Implementer:** the ‘doer’	A reliable worker who puts the ideas into action, although they may lack flexibility.
**Completer finisher:** the ‘details’ person	Is conscientious in completing the job, and pays attention to detail.
**Resource investigator:** the ‘networker’	Sources information and resources, acts as the group’s ‘ambassador’, although enthusiasm may fade during the project.
**Shaper:** the ‘driver’	Keeps the project moving, enjoys the action, but can upset others as they push through the ideas.
**Teamworker:** the ‘peacemaker’	Assists with diplomacy and helps keep the team working effectively, although they can be indecisive.
**Specialist:** the ‘expert’	Provides expert knowledge, although their input may be restricted to their own specialised area.

### Effective leadership

Leaders need to have good time management and organisational skills, the ability to network professionally, display political nous and most importantly, they need to have strong communication skills [4, 20, 21]. Ready acceptance of feedback and self-awareness are important in development of leadership skills [20, 21]. Behaviour, habits and biases can be deliberately corrected by utilising received feedback. Although there is not one set of qualities that apply to being an effective leader, certain competencies are valued and contribute to the leadership model in different ways [5]. Leadership competencies relevant for all health professional educators are outlined in Table 4 [3].

**Table 4:** Leadership competencies for health professional educators (adapted from Oates, 2012) [3]

**Knowledge of leadership concepts**	
• This includes theoretical background, organisational structure, and leadership development of others.	
**Motivator, mentor and facilitator**	
• Integrity should be shown in motivating and encouraging others instead of controlling situations.	
• Through excellence in role modelling, and careful delegation, future leaders are developed, and succession planning can occur.	
**Communicator**	
• Good communication entails consistent messages through various methods over time.	
• Communication by leaders is required at all levels: to senior management, administrators, team members, and to patients.	
• Communication should always be respectful, and acknowledge the input and achievements of others.	
• Networking, facilitating groups, effective listening and feedback skills.	
**Ability to set direction and lead change**	
• Understand the environment, set goals, change management, decision making.	
**Leadership presence**	
• The ability to assume a leadership role in various settings, share your opinion with confidence, and communicate and engage with others.	
**Team leader, team player, team-building**	
• A good leader is not only a team leader, but also a team player, who values and seeks the opinions of others.	
• Leaders are involved in teaching, coaching and mentoring, holding team members accountable, and undertaking performance appraisals.	
• Conflict resolution skills are needed in leadership roles. The views and abilities of all parties should be respected.	
• Group problem-solving, conflict management, contributions to team processes and development.	
**Healthcare education research skills**	
• Although time may not permit involvement in educational research, a good leader will have the ability to critically appraise research, and an understanding of the value of research.	
**Business skills**	
• Human Resource management, work flow, budgeting, effectiveness evaluation, business plan development.	
• Reduction of waste and inefficiencies.	
• Financial management skills, including resource allocation, reduction in variation of clinical practice to reduce costs, and increase provisions for clinical care.	
**Self-management**	
• Time management, work-life balance.	
**Ability to develop others**	
• Coaching, motivating, interpersonal effectiveness.	

### Language of leadership

Just as education and healthcare organisations have evolved, so too has the team leader. The role of the modern leader reinforces the tenets of stepping forward, collaborating and contributing. This role involves encouraging others by practising followership, and lending meaningful support to other leaders. As already stated, when it comes to leadership, excellent communication skills are a must. In order for successful communication to occur, both the sender and receiver must understand the message. This means that active listening is just as important as active talking [22]. Language used needs to be [22]:

*Clear*

- Communicate with clarity of your purpose and the role of others

*Stimulating*

- Deliver messages in a powerful, inspiring and dramatic way

*Congruent*

- Lead by example and walk the talk

*Include active listening*

- Acknowledge what has been communicated, and use questioning skills
- Show that you value others and their contributions

### Challenges for leaders in healthcare education

**Fig. 1:**
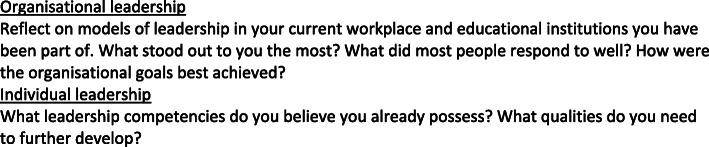
Reflection task

There are a number of unique challenges in healthcare education. Healthcare education is delivered across professional disciplines, and notably, across organisational boundaries, involving universities, hospitals, and healthcare services. In turn, these organisations are bound by their own systems, structures, policies, cultures and values. At some point, most leaders in healthcare education need to make a decision about their leadership direction, and whether it lies predominantly in higher education or the clinical setting; and whether it lies in undergraduate education or postgraduate education. It can be difficult to merge roles between organisations, and McKimm (2004) has identified a number of issues and challenges specific to health education leaders, outlined in Table 5 [22, 23]. Throughout a career, it may be necessary to maintain an awareness of available opportunities within organisations, and match these to the required experiences and capabilities [22, 23] (see Fig. 1).

**Table 5:** Issues and challenges of health education leaders (adapted from McKimm, 2004) [22, 23]

***Personal issues***	
• It can be difficult to maintain an appropriate work-life balance, particularly for those with family responsibilities.	
• Managing both clinical and academic careers is difficult.	
***Organisational and cultural issues***	
• In order to succeed, leaders need to understand the culture of their own organisation.	
• Some healthcare disciplines may better facilitate the demands of both clinical and academic life.	
***Balancing competing agendas***	
• Dual demands of the higher education sector, which is highly accountable, and healthcare systems, with rapid change, may be stressful for healthcare education leaders.	
***The wider agenda***	
• Education leaders need to have an awareness of the wider healthcare and education agendas, and help drive new issues, such as interprofessional learning and collaboration. They need to help promote diversity and innovation in leadership.

### Development of leadership skills

Workforce data indicates that many experienced clinicians and healthcare educators will retire over the next ten years [24, 25]. The need for effective succession planning and leadership training is well recognised [25–27], with a current shortage of emerging leaders moving into leadership roles. Effective leaders need to be nurtured and supported by the organisations in which they are educated, train and work [6]. As a learned skill, the topic of leadership is gathering momentum as a key curriculum area. Leadership development, assessment and feedback are necessary throughout the education and training of health professionals. Aspiring and current leaders can be identified, trained and assessed through formal leadership development programs, and through supportive organisational cultures. This requires embedding leadership training programs, opportunities for leadership practice, and promotion of professional networks within and beyond the organisation. The importance of mentorship within healthcare education is well recognised, offering a means to further enhance leadership and engagement within the workforce [28].

While many are *assigned* as leaders through their job title, it is important to identify, support and develop *emerging* leaders [2]. Leadership consists of a learnable set of practices and skills that can be developed by reading literature and attending leadership courses [29]. Additionally, investment in the social capital of organisations, fostering interprofessional learning and communication in the work setting, and collaboration across organisations assists in leadership development. Developing leadership skills is a life-long process [21]. Resources and opportunities should be considered to assist in the development of leadership skills. Some examples include:

- Reading about leadership e.g. theories on leadership styles
- Attending leadership training workshops
- Participating in mentorship programs either as mentee or mentor
- Joining small group seminars on leadership development
- Accepting more responsibilities when required, or when opportunities arise.

### Process for effective leadership

A title is not required to enable effective leadership. Leadership may occur in everyday work, and occurs in collaboration with other professionals within the education and healthcare systems. For example, leadership in teaching, administration, research, and/or excellence in clinical practice.

Leadership roles include the important concept of management of both personal and professional practice. Priorities need to be set and time managed to integrate work and personal life. Tools can be used to stay organised, and deliberately manage busy schedules. Effective delegation may be used to share the work of new projects:

- Organisation to ensure an understanding of tasks, priorities and deadlines
- Establish steps and a sequence to achieve the desired outcomes
- List required resources, considering the competencies of individual team members, and match tasks appropriately (also consider skill development needs)
- Communicate with team members, monitor progress in activities and provide guidance to team members.

## Conclusion

Leadership competencies, and the incorporation of leadership development as part of curricula, are identified as important across all health professions, including allied health, nursing, pharmacy, dentistry, and medicine, in meeting the needs of healthcare in the twenty-first century [30]. With an increase in interprofessional teams and an emphasis on collaboration, more effective outcomes are achieved [5]. Healthcare education leaders are required to work effectively and collaboratively across discipline and organisational boundaries, where titles are not always linked to leadership roles, but may occur in everyday work. Good leadership also means knowing when, and how to support others in their endeavours. Provision of opportunities for leadership development is crucial in improving education sectors and health services, and effecting change. The future belongs to healthcare education leaders who demonstrate excellence in teamwork, clinical skills, patient centred care [3], and responsibly balance accountability with autonomy.

### Take-home message

**Table:** 

• Titles are not always linked to leadership roles. • The role of today’s leader requires stepping forward, collaborating and contributing. • A good leader is a good team player who values and seeks the opinions of others. • Leadership requires clear, respectful communication that acknowledges the input and achievements of others.	

## Data Availability

Not applicable.

## Abbreviation

HR: Human Resources

## References

[1] Bennis W, Nanus (1985). Leaders: the strategies for taking charge.

[2] Northouse P (2011). Leadership: theory and practice.

[3] Oates K (2012). The new clinical leader. J Paediatr Child Health.

[4] Laverack G (2014). The pocket guide to health promotion.

[5] Clarke J, Patole S (2015). Management and Leadership – A Guide for Clinical Professionals.

[6] Swanwick T, McKimm J (2011). What is clinical leadership and why is it important. Clin Teach.

[7] Byrman A, Clegg SR, Harvey C, Nord WR (2006). Leadership in organisations. Handbook of Organisational studies.

[8] Westley F, Minztberg H (1989). Visionary leadership and strategic management. Strateg Manag J.

[9] Bass B, Avolio B (1994). Improving Organisational effectiveness through transformational leadership.

[10] Coulson A (1986). The managerial work of Headteachers.

[11] Alimo-Metcalfe B, Alban-Metcalfe J, Storey (2004). Leadership in public organisations. Leadership in Organisations: current issues and key trends.

[12] McKimm J, Swanwick T (2007). Educational leadership.

[13] Levi D (2011). Group dynamics for teams.

[14] Burgess A, McGregor D, Mellis C (2014). Applying guidelines in a systematic review of team-based learning in medical schools. Acad Med.

[15] Pearce CL, Manz CC, Sims HP (2009). Where do we go from here? Is shared leadership the key to team success?. Orgnisational Dynamics.

[16] Morgeson FP, DeRue DS, Karan EP (2010). Leadership in teams: a functional approach to understanding leadership structures and processes. J Manag.

[17] Kozlowski SWJ, Gully SM, Salas E, Cannon-Bowers JA (1995). Team leadership and development: theory, principles, and guidelines for training leaders and teams.

[18] Tuckman B (1965). Developmental sequence in small groups. Psychol Bull.

[19] Belbin RM (1991). Management teams: why they succeed or fail.

[20] Glover Takahshi S, Abbott C, Oswalk A, Frank JR (2015). CanMEDS Teaching and Assessment Tools Guide. Royal College of Physicians and Surgeons of Canada.

[21] Clawson J (2006). Level three leadership: Getting below the surface (3rd ed.).

[22] McKimm J. Developing tomorrow’s leaders in health and social care education. Case studies in leadership in medical and health care education. Special report 5. Newcastle-upon-Tyne: Higher Education Academy, Medicine Dentistry and Veterinary Medicine, 2004.

[23] McKimm J, Swanwick T (2011). Leadership development for clinicians: what are we trying to achieve?. Clin Teach.

[24] Norcini JJ, Banda SS (2008). The global health workforce shortage: role of surgeons and other providers. Adv Surg.

[25] Kim T (2012). Succession planning in hospitals and association with organizational performance. Nurs Econ.

[26] Swanwick T, McKimm J (2012). Clinical leadership development requires system-wide interventions, not just courses. Clin Teach.

[27] Matthews JH, Morley GL, Crossley E, Bhanderi S (2017). Teaching leadership: the medical student society model. Clin Teach.

[28] Burgess A, van Diggele C, Mellis C (2018). Mentorship in the health professions: a review. Clin Teach.

[29] Burgess A, Dornan T, Clarke A, Menezes A, Mellis C (2016). Peer tutoring in a medical school: perceptions of tutors and tutees. BMC Medical Education.

[30] Druker PF (2004). What makes an effective executive?. Harv Bus Rev.

